# Atherosclerosis: The Involvement of Immunity, Cytokines and Cells in Pathogenesis, and Potential Novel Therapeutics

**DOI:** 10.14336/AD.2022.1208

**Published:** 2023-08-01

**Authors:** Chang Su, Yongzheng Lu, Zeyu Wang, Jiacheng Guo, Yachen Hou, Xiaofang Wang, Zhen Qin, Jiamin Gao, Zhaowei Sun, Yichen Dai, Yu Liu, Guozhen Liu, Xunde Xian, Xiaolin Cui, Jinying Zhang, Junnan Tang

**Affiliations:** ^1^Department of Cardiology, First Affiliated Hospital of Zhengzhou University, Zhengzhou, Henan, China.; ^2^Key Laboratory of Cardiac Injury and Repair of Henan Province, Zhengzhou, Henan, China.; ^3^Henan Province Clinical Research Center for Cardiovascular Diseases, Zhengzhou, Henan, China.; ^4^School of Medicine, The Chinese University of Hong Kong, Shenzhen, Guangdong, China.; ^5^Institute of Cardiovascular Sciences, Peking University, Beijing, China

**Keywords:** atherosclerosis, pathogenesis, inflammation, molecular therapy, cellular therapy

## Abstract

As a leading contributor to coronary artery disease (CAD) and stroke, atherosclerosis has become one of the major cardiovascular diseases (CVD) negatively impacting patients worldwide. The endothelial injury is considered to be the initial step of the development of atherosclerosis, resulting in immune cell migration and activation as well as inflammatory factor secretion, which further leads to acute and chronic inflammation. In addition, the inflammation and lipid accumulation at the lesions stimulate specific responses from different types of cells, contributing to the pathological progression of atherosclerosis. As a result, recent studies have focused on using molecular biological approaches such as gene editing and nanotechnology to mediate cellular response during atherosclerotic development for therapeutic purposes. In this review, we systematically discuss inflammatory pathogenesis during the development of atherosclerosis from a cellular level with a focus on the blood cells, including all types of immune cells, together with crucial cells within the blood vessel, such as smooth muscle cells and endothelial cells. In addition, the latest progression of molecular-cellular based therapy for atherosclerosis is also discussed. We hope this review article could be beneficial for the clinical management of atherosclerosis.

## 1. Introduction

Atherosclerosis has gradually become a worldwide health issue over the past decades, which often results in artery-clogging, myocardial infarction (MI) and other fatal cardiovascular diseases (CVD). The Global Burden of Disease (GBD) Study 2019 showed that the number of CVD cases worldwide increased from 271 million in 1990 to 523 million in 2019 [1]. In fact, cardiovascular disease is the leading cause of mortality accounting for 17.9 million deaths in 2019 [2]. Unhealthy lifestyles such as smoking, the rising popularity of fried foods, increasing stress and inactive physical exercises have affected the general population’s health and contributed to the development of diseases like diabetes, hypertension, obesity, and metabolic syndrome, which promote endothelial injury and subsequently increase the risk of atherosclerotic-related CVD (ASCVD) [3].

Current clinical management for atherosclerosis often includes pharmaceutical and surgical interventions (Table 1). As the most common drug, statins can decrease blood lipid levels, promote plaque stability, and inhibit lipid accumulation in atherosclerotic plaque [4], which has been widely used in clinics to reduce artery clogs. Meanwhile, other drugs such as PCSK9 inhibitors and antiplatelet medicine have also been applied clinically, showing sufficient therapeutic effects. However, the disadvantages of pharmaceutical treatment include hepatic injury, bleeding risk, and arrhythmia, resulting in inconsistent clinical outcomes [5]. Surgical interventions are normally used at a later stage. Approaches such as angioplasty, endarterectomy, bypass surgery and atherectomy, are applied worldwide. Although the surgical technique can remove the atherosclerotic plaque effectively with an improved short-term therapeutic effect, the potential complications and risks associated with the anesthesia and surgery, the strict requirement for medical facilities, and the failure to prevent the reoccurrence of atherosclerosis may result in an unsatisfactory long-term outcome [6]. Therefore, new therapies are still being pursued. To better address those challenges, recent research interest has shifted to focus on cellular and molecular technology such as gene editing and nanotechnology to target the cellular response during the development of atherosclerosis, aiming to slow and even prevent atherosclerotic progression.

**Table 1:** Medical treatment for atherosclerosis in clinical.

Medical treatment		Typical	Mechanism	Side effect
**Statins**		Atorvastatin Rosuvastatin Simvastatin	Inhibit HMG-CoA, the rate-limiting enzyme in cholesterol synthesis. Activates hepatic LDL receptors. Improve LDL receptor-mediated plasma LDL clearance [234]	Muscle Symptoms (Myalgia, Myositis,Rhabdomyolysis) [235]; SINAM [236]; Diabetes [237]; Liver failure [238]
**PCSK9 inhibitor**		Evolocumab	Targeting PCSK9 to increase LDL receptor re-circulation [239]	Allergic reactions [240]
**Betas**		Fenofibrate	Activate PPARα. Induce the expression of lipoprotein esterase. Remove triacylglycerol-rich lipoproteins from plasma, and increases HDL levels [241]	Hcy increase [242] Pancreatitis [243] Pulmonary embolism [244]
**Cholesterol absorption inhibitor**		Ezetimibe	Inhibit cholesterol transport protein Niemann Pick C1 like 1 protein [245]	Digestive symptoms
**Antioxidants**		Probucol	Reduce ROS [246]	Digestive symptoms Arrhythmia [247]
**Anti-platelet**	TXA2 inhibitor	Aspirin	Inhibit COX to block down TXA2 synthesize [248]	Bleeding Renal failure Hemolysis [249]
	ADP P2Y12 receptor inhibitor	Clopidogrel Ticagrelor	Bind to ADP receptors on the platelet membrane and prevents exposure of the binding site of GPIIb/IIIa receptors, inhibit the aggregation of platelets [250]	Bleeding TTP
	GPI	Tirofiban	Inhibit GPIIb/IIIa receptor [251]	Bleeding TTP
	Platelet adenosine cyclase stimulant	Prostacyclin	Activate adenylate cyclase, increases the concentration of cAMP in platelets [252]	Hypotension Tachycardia
	5-HT receptor inhibitor	Sarpogrelate	Inhibit 5-HT2 receptor, reduce platelet aggregation [253]	Bleeding TTP

LDL: Low-Density Lipoprotein; HDL: High-Density Lipoprotein; SINAM: Statin-induced Necrotizing Autoimmune Myopathy; TTP: Thrombotic Thrombocytopenic Purpura; GPI: Glycoprotein IIb/IIIa Inhibitor.

Fundamentally, during the occurrence of atherosclerosis, endothelial cells on intima wall of the artery are activated or damaged, resulting in the infiltration of lipids and lipoproteins such as low-density lipoproteins (LDLs), as well as the migration of smooth muscle cells (SMCs) into intima [7]. Following the injury, the necrosis of ECs induces the inflammatory factors secretion, leading to the migration, activation, and aggregation of monocytes at the lesion [8]. The activated monocytes could induce the apoptosis of ECs, resulting in further disruption to the endothelial layer. Meanwhile, LDLs become oxidized (ox-LDLs) and aggravate artery injury. In addition, the SMCs and monocytes accumulated at the intima take up the ox-LDLs and then transform into foam cells, which is the initial step of atherosclerotic plaque development [9]. Interestingly, recent studies indicate that endothelial injury might not be necessary for the development of atherosclerosis, and some stimulating factors, such as changes in the shear stress, could also promote the formation of fatty streak [10]. Hypothetically, if the cellular response could be interfered with, the pathological progression of atherosclerosis would be prevented. In fact, recent research has demonstrated the promising preclinical results with using cellular and molecular technology to prevent atherosclerotic progression.

Although current literature has summarized some important cellular responses such as some immune cells (namely macrophages and neutrophils) or SMCs, a gap still exists to provide an overall picture of all the relevant cells, such as B-cells, T-cells, platelets, and granulocytes, as well as the recently developed cellular level-based technologies. Hence, a review to reflect the progression of cellular and molecular-based therapy in atherosclerosis can be timely and beneficial. Herein, this paper reviews the inflammatory pathogenesis during the pathological progression of atherosclerosis on a cellular level. In addition, a comprehensive analysis of different cellular responses, including ECs, SMCs, and all the immune cells, is discussed. Furthermore, by providing the latest progression of the molecular-cellular based therapy in atherosclerosis treatment, we hope this article could provide some insightful information for clinicians to reconsider the clinical management of atherosclerosis.

## 2. Cellular response during the development of atherosclerosis

The fundamental mechanism of atherosclerosis development involves complicated cellular responses after the endothelial injury, including the activation, migration and accumulation of immune cells, apoptosis and damage of ECs and infiltration and transformation of SMCs.

### 2.1 Immune cells

Atherosclerotic lesions often accumulate at the artery intima, consisting of cells, lipids, connective tissue, and cell debris. Recently, studies indicated that most types of cells within atherosclerotic plaques are immune cells, together with ECs and SMCs. Chronological predecessors of atherosclerotic lesions are fatty streaks mainly composed of foam cells (lipid-laden macrophages/ SMCs) [11], supplemented with T cells, dendritic cells, and mast cells. Often fatty streaks are prevalent in the younger population without symptoms [12, 13]. However, fatty streaks can further develop into atherosclerotic plaques with more complicated structures.

Atherosclerotic plaques have a core composed of macrophage foam cells, dead cells, and lipids, surrounded by a layer of SMCs and collagen-enriched extracellular matrix. T cells and macrophages have been found throughout the whole lesions. Interestingly, in specific regions, including the area where lesions grow and the interface between the core and outer SMCs layer, an increased number of immune cells have been observed [14]. More importantly, many immune cells are active and continuously secreting proinflammatory cytokines during the disease progression [15]. As a result, atherosclerosis, once known as a lipid accumulation disease, has now been considered as an inflammatory disease. Therefore, the immune cells’ response is vitally important to understand the pathological development of atherosclerosis.

#### 2.1.1 Innate Immune Cells

##### 2.1.1.1 Monocytes and Macrophages

Macrophages are the dominated cells located in atheromatous plaques, playing a critical role during atheromatous plaques growth. Under normal physiological conditions, macrophages contribute to the germinal process of blood vessels. Leid found that CCR2^-^macrophages derived from the yolk membrane in mice can promote the development of primitive coronary arteries by secreting IGF1 and IGF2 [16]. However, the abnormal differentiation of macrophage may lead to the occurrence of atherosclerosis. Often, common risk factors for atherosclerosis, such as hyperglycemia [17] and hyperlipidemia [18, 19], can cause bone marrow hematopoiesis. Hypercholesterolemia inhibits lipid efflux of hematopoietic stem cells, leading to abnormal myelopoiesis and differentiation [18]. ABCA1^-/-^ABCG1^-/-^ mice present bone marrow hyperplasia and elevated leukocytes due to the blocked cholesterol efflux [19]. When ApoE ^-/-^ mice were fed with a high cholesterol diet, an increased number of the monocyte count was observed [20]. Cholesterol induces the proliferation of myeloid progenitors, granulocyte, and macrophage progenitors, which accelerates monopoiesis. In addition, smoking [21], lack of exercise [22] or sleep [23] also have similar effects on bias myeloid cell hematopoietic.

**Figure 1.**
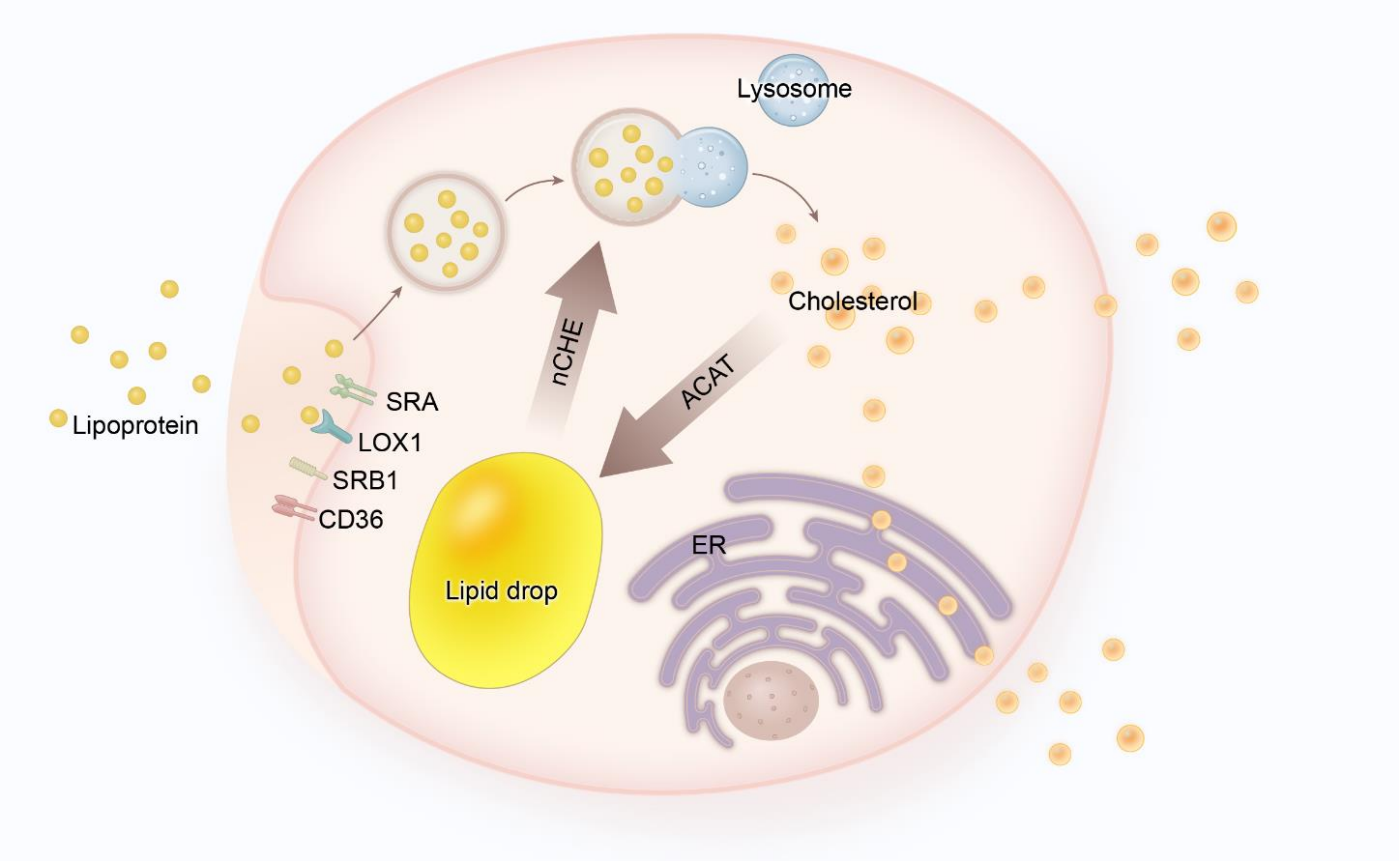
**Lipid metabolism in macrophages**. After entering macrophages, lipoproteins are converted into cholesterol in lysosomes. After that, cholesterol can flow out of the cell through the membrane or be converted into lipid droplets by ACAT for storage. In fact, ACAT and nCHE activities control the transformation of macrophages into foam cells.

On the early stage of atherosclerosis, macrophages are recruited to the vascular intima in response to chemokines or integrin. A study demonstrated that blocking receptors of macrophages could reduce plaque burden, proving the involvement of macrophages in the pathogenesis of atherosclerosis [24-26]. After the recruitment to the lesion area, macrophages form the lipid core under the complex pathological environment. The imbalance of lipid metabolism in macrophages becomes a key factor during this process. In addition, lipoproteins enter the sub-endothelial space, further aggravating the vascular endothelial injury [27]. More importantly, these lipoproteins are susceptible to various modifications, such as oxidation or crystallization [28]. Modified lipoproteins have a strong proinflammatory effect and can activate endothelial cells to secrete several inflammatory factors and chemokines (IFN-γ, TNF-α, MCP-1, et al.) directly, resulting in the further recruitment of monocytes [29]. Recruited monocytes differentiate into macrophages and then internalize oxidized lipoproteins. Studies have demonstrated that the internalized lipoproteins are regulated by several receptor-mediated signaling pathways, including scavenger receptor A (SRA), oxidized low-density lipoprotein receptor-1(LOX-1), scavenger receptor class B member 1 (SRB1) and CD36 [27]. Normally, after lipoproteins enter the macrophages, lysosomes digest internalized lipoproteins and then release free cholesterol [30] which further reaches the plasma membrane, leading to the outflow from the cell membrane or endoplasmic reticulum (ER) [31, 32]. Cholesterol retained inside the cell is esterified by acyl-coenzyme a-cholesterol acyltransferase (ACAT) and stored as lipid droplets [33, 34]. These stored cholesterol esters can be mobilized by neutral cholesteryl ester hydrolase (nCEH) and delivered to lysosomes for further processing. nCEH is considered a rate-limiting enzyme in foam cell formation. Sekiya found that ablation of nCEH accelerates atherosclerosis in mice, which indicates the role of nCEH in preventing foam cell formation [35]. Accumulation of cholesterol activates liver-X-receptor (LXR)/retinoic-X-receptor (RXR) heterodimeric transcription factor and upregulates the expression of ABC transporters (ABCA1, ABCG1) [36], which mediate the transfer of free cholesterol to ApoA1 in order to form high-density lipoprotein (HDL) [37]. However, due to the imbalance in the lipid intake and efflux, macrophages start to internalize overflowed lipids and further transform into foam cells, which leads to the loss of their migratory capacity [38, 39]. As a result, foam cells gradually accumulate in the lesion area and further promote the progression of atherosclerosis. The lipid metabolism process in macrophages is shown in Figure 1.

**Figure 2.**
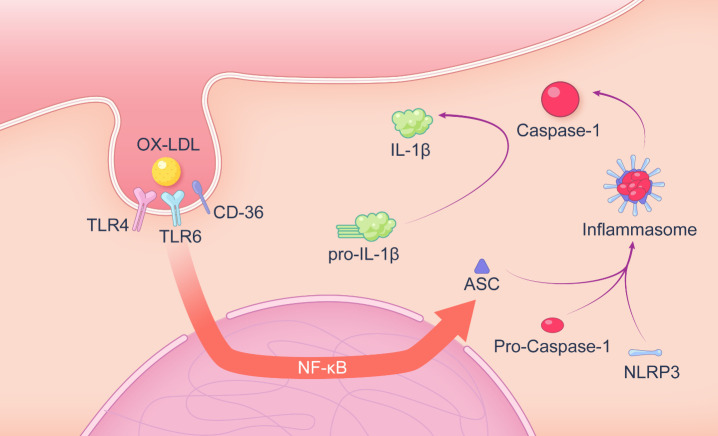
**The intracellular production mechanism of macrophage secreted IL-1β**. Oxidized low-density lipoprotein binds to CD36, TLR4, and TLR6 complexes. Accumulation of lipids activate the macrophage NF-κB pathway, triggering the transcription of NLRP3 inflammasomes and pro-IL-1β. NLRP3 inflammasome can release caspase1 to cleave pro-IL-1β to IL-1β.

During the progress of atherosclerosis, foam cells secrete several inflammatory factors (such as several chemokines, various cytokines and reactive oxygen species) and matrix-degrading proteases. Among factors above, IL-1β is considered to play a key role in atherosclerotic progression [40, 41]. The intracellular production mechanism of IL-1β is shown in Figure 2. Besides, Il-1β has been found to be produced extracellularly through a non-caspase-1 pathway, which may relate to MMP9, neutrophil PR3 (proteinase 3) and granzyme A [42]. The coding genes of IL-1family are different, but their physiological functions are similar due to the same downstream receptor IL-1R [43]. IL-1β can increase the expression of related genes in downstream cells, such as inducing COX-2 to produce prostaglandins and increasing iNOS activity to regulate the NO production pathway [44]. IL1-β also increases the expression of adhesion factors (T cell receptor-1, VCAM-1, et al.), which results in the accumulation of inflammatory cells [45, 46]. There is a positive feedback loop *in vivo* for IL-1, as the elevated IL-1 gene expression in the downstream cells allows a further increase in IL-1 production following IL1-R activation [47]. IL-1 can also increase the secretion of IL-6, which has been demonstrated to have a strong prothrombotic substance secretion function [48]. Foam cells secrete IL-1 to aggravate local inflammation, and the aggravation of local inflammation and foam cell apoptosis further worsens the microenvironment in the lesion area [49]. Necroptosis of foam cells releases cellular contents, inducing the inflammatory response on other cells [49]. Accumulation of excessive free cholesterol activates the NLRP3 inflammasome [50], which may also interfere with the function of the endoplasmic reticulum (endoplasmic reticulum stress), leading to the inflammation reaction and apoptosis over time [51, 52]. Necrosis and apoptosis of foam cells reduce plaque stability, giving rise to plaque rupture and thrombosis, which contribute to acute myocardial infarction and stroke.

##### 2.1.1.2 Natural Killer cells

NK cells, differentiated from bone marrow lymphoid stem cells, are the first line of immune defense in the body. Due to the difference between CD56 and CD16 expression, NK cells in peripheral blood are divided into two subtypes with different physiological functions. CD56^bright^NK cells secrete various cytokines, such as IFN-γ [87]. CD56^dim^NK cells, on the other hand, express the low-affinity IgG receptor FcγRIIIa (CD16) and exhibit cytotoxicity [88].

Chemokines secreted by cells related to atherosclerosis progressions, such as monocyte chemotactic proteins1 (MCP-1) and CX3CL, can promote the migration of NK cells to plaques. It has been confirmed that the amount of NK cells rises in atheromatous plaques, especially in unstable plaques [89]. In addition, the expression of killer activating receptor NKG2C on NK cells surface increase in carotid atheromatous plaques [90]. Relevant clinical studies have also found that high circulating NK cells amount is associated with severe atherosclerosis [91, 92], which all indicate that NK cells contribute to the pathological progression of atherosclerosis. Interestingly, ox-LDL, the contributor to the foam cell formation, could induce apoptosis of NK cells, however, antioxidant serotonin (5-HTP) can protect NK cells from apoptosis [93]. Normally, ox-LDL produces 7β-hydroxycholesterol (7βOH), which disrupts the lysosomal membrane of NK cells and leads to the apoptosis of NK cells and releases ROS, further aggravating other immune cells' activation [94]. The specific roles of NK cells played in the formation of atherosclerosis are still being investigated. In a recent study, Nour-Eldine found that in Ncr1^iCre/+^R26l^sl-DTA/+^ mice and Noé mice, neither NK cells deficiency nor hyperreactivity affected the development of atherosclerosis. However, NK cell deficiency can prevent atherosclerosis occurrence under the conditions of lentiviral injection, suggesting that NK cells may affect the development of atherosclerosis by participating in chronic inflammatory responses under specific conditions [95]. A study has also found that LPS can increase NK cells and NKT cells in ApoE ^-/-^ mice but not in ApoE ^-/-^ CD1d ^-/-^ mice; meanwhile, the atherosclerosis area in mice is not significantly increased [96]. This suggests that NK cells and NKT cells may act synergistically in the development of atherosclerosis. Still, the specific mechanism of NK cells in atherosclerotic diseases remains elusive.

**Figure 3.**
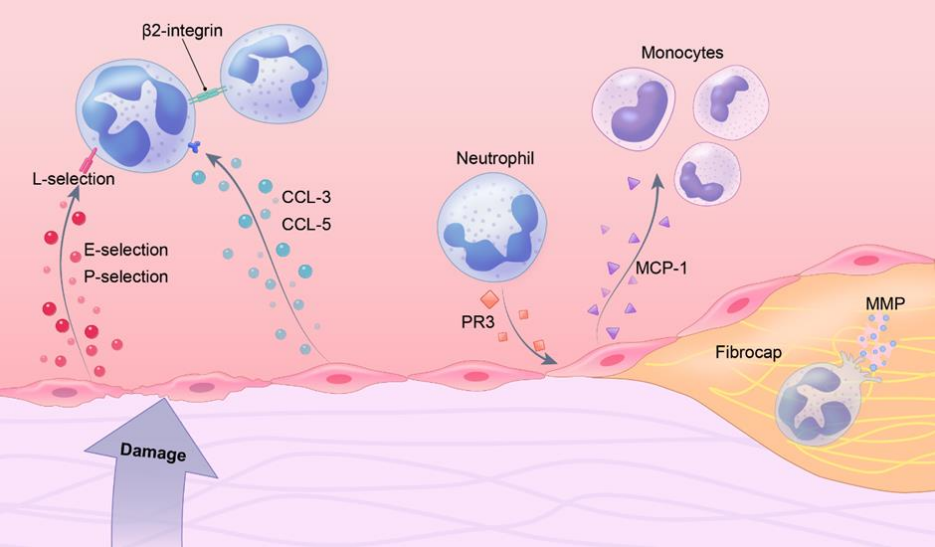
**Neutrophils are involved in the whole process of atherosclerosis**. Initially, neutrophils are recruited to the lesion area under the effect of cytokines secreted by injured endothelial cells. Later, accumulated neutrophils in the injured area can secrete PR3 and promote endothelial cells secrete chemokines, which further leads to the recruitment of other inflammatory cells. In the end stage of atherosclerosis, neutrophils interior the plaque secretes MMP continuously, which leads to the destruction of the microenvironment in the plaque and the decline of plaque stability.

##### 2.1.1.3 Granulocytes

Granulocytes, mainly eosinophils, basophils, and neutrophils, influence the progression of atherosclerosis by participating in inflammatory responses.

Neutrophils have been widely referred to as the whistles of the inflammatory response, often being the first cells to reach the site of inflammation. In atherosclerosis, injured endothelial cells secrete e-selectin and p-selectin, which binds to L-selectin on the surface of neutrophils, allowing neutrophils to recruit to the surface of endothelial cells [103]. CCL3 and CCL5 secreted by endothelial cells activate neutrophils to express β_2_ integrin, promoting neutrophils adhesion and aggregation [104]. Activated neutrophils can induce endothelial cells to secrete MCP-1 by secreting protease 3 (PR3), as a result, recruit monocytes to the lesion site and promote macrophages polarization [105]. Under normal physiological conditions, neutrophils can be cleared by macrophages to stop the further inflammatory response. In atherosclerotic lesion site, however, neutrophils cannot be properly cleared, which results in continued secreting MMPs. Persistent MMPs lead to dissolving of the fibrous cap and extracellular matrix, giving rise to the reduced stability of atherosclerotic plaques and increased risk of rupture [106]. Neutrophils are involved in the whole process of atherosclerosis, which is shown in Figure 3.

In addition to neutrophils, clinical evidence also suggests that peripheral blood eosinophils are associated with the degree of atherosclerosis [107]. However, the underlying mechanism is still unclear. It is hypothesized that chemokine expression during atherosclerosis development theoretically led to the accumulation of eosinophils, which contains proteases such as major basic protein (MBP) or eosinophil peroxidase (EPX) that exacerbate local inflammatory responses and tissue damage. Marx found that eosinophils increased endothelial von Willebrand factor expression and cause platelet adhesion [108]. Thus, eosinophils activate the attached platelets by forming MBP - modified eosinophil extracellular traps (EET).

Basophils are inflammatory immune cells mediated by IgE. At present, limited studies are looking into how basophils affect the progression of atherosclerosis.

Mast cells can be regarded as a special kind of basophil, which are differentiated from the same stem cell but mature and distributed at different locations [109]. Considering the similarity between them, the pathogenesis of basophils may be similar to mast cells in atherosclerosis. Mast cells store many secretory granules in the cytoplasm and are involved in several immune responses in the human body. Histamine, neutral proteases, chemokines, and cytokines can be released into extracellular after mast cell activation. That is induced by factors such as allergen related IgE, endothelin and ROS.

Mast cells can bind to chemokines CCL11 and CXCL1, highly expressed in atheromatous plaque areas, through chemokine receptor 3 (CCR3) on the cell membrane, resulting in enrichment degranulation of mast cells in atherosclerosis [110]. IgE secreted by immune cells in the plaque area can also bind to FcepsilonRI receptors on mast cells' surface and prompt degranulation [111]. Mast cells aggravate the pathological progression of atherosclerosis by exerting their secretory function. Chymase, a chymotrypsin-like serine protease secreted from mast cells, can convert angiotensin I to angiotensin II (Ang II), contributing to the development of atherosclerosis [112]. As a member of the neuro-humoral pathway of blood pressure regulation, Ang II regulates the vessel wall, leading to the elevated expression of e-selectin, ICAM-1 and VCAM-1 in endothelial cells, and the enhanced recruitment of macrophages [113]. Furthermore, Ang II could upregulate LOX-1 in macrophages and endothelial cells, resulting in the increased ox-LDL uptake and subsequently deteriorated cellular function [114]. Ang II can also upregulate NADPH in endothelial cells and lead to NADPH-dependent ROS formation [115]. Most directly, ROS can oxidize LDL to ox-LDL. Secondly, ROS can reduce the NO bioavailability of endothelial cells. It is expected that ROS reacts with NO to produce ONOO- and inactivate NO, with reduced NO production due to the downregulated eNOS pathway [116]. Furthermore, the increased concentration of eNOS inhibitor asymmetric dimethylarginine may contribute to the declined NO release from endothelial cells [117]. In addition, chymase can also degrade vessel collagen and induce SMCs and ECs apoptosis [118]. HDL degradation is also a consequence of chymase release which interferes with cholesterol metabolism and then promotes foam cells formation [119]. Apart from chymase, other chemokines secreted by mast cells (CXCL1, CXCL2, MCP-1, CCL5/RANTES) could contribute to inflammatory cells aggregating to plaque areas [120, 121] and magnifying the immune response. Histamine secreted by mast cells increases vascular permeability, facilitates inflammatory cell infiltration, and reduces plaque stability as a result. Inflammatory factors released by mast cells, such as TNFα and IFNγ [121, 122] can promote local inflammation. Moreover, their secreted growth factors, such as VEGF, can increase angiogenesis within the plaque and further promote plaque growth [123]. Taken together, mast cells play an essential role during the pathological development pf atherosclerosis.

##### 2.1.1.4 Dendritic Cells

Dendritic cells are antigen presenting cells (APCs), whose main physiological function is to recognize antigens and present them to T cells for the activation of specific immunity. Under normal physiological conditions, immature DCs distribute in human peripheral blood. Following the TLR receptor activation and antigen capture, DCs quickly migrate to the lymphatic system and mature to achieve their function. However, during the development of atherosclerosis, the normal physiological function of DCs is disrupted, which aggravates the progression of atherosclerosis. At the early stage of the disease, DCs can phagocytose lipids which is helpful for antigen-presenting. However, in hyperlipidemia, CCR7 of DCs is not sufficiently upregulated, and the migration function of DCs is seriously impaired, leading to local deposition of foam cells transformed from DCs [124]. Normally, dendritic cells activate Treg cells in non-inflammatory lymph nodes. However, due to the impaired migration, T cells also activate in an inflammatory environment, which results in a decrease in protective Treg cells and exacerbates atherosclerosis [125]. In addition, mature DCs lack the ability of efferocytosis, which contributes to secondary necrosis in the lesion and exacerbating local inflammation [126]. Overall, dendritic cells have a similar role in atherosclerosis as in other autoimmune diseases, opening a new target potential.

#### 2.1.2 Adaptive immune cells

##### 2.1.2.1 T cells

In recent years, the importance of macrophages in the pathogenesis of atherosclerosis has been increasingly appreciated. In contrast, T cells, another important immune cell, have been overlooked.

**Figure 4.**
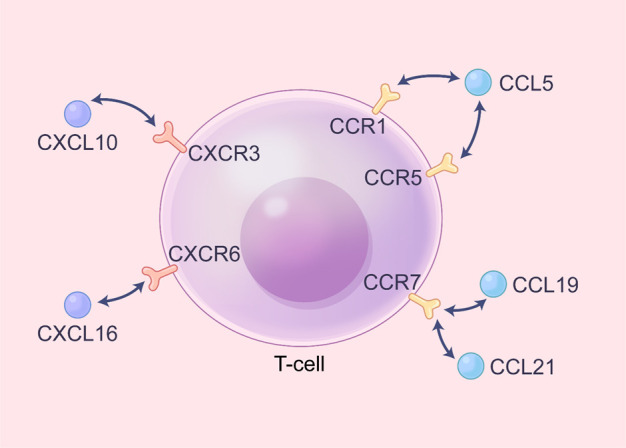
**Chemokines and their receptors for T cell homing in atherosclerosis**. At the atherosclerosis site, ECs, SMCs and other inflammatory cells (macrophages, mast cells, et al.) secrete several chemokines (CCLs and CXCLs), which can move the disease process forward by targeting specific receptors (CCRs and CXCRs) on the surface of T cells and allowing T cells to recruit to the lesion site.

Given that atherosclerosis is an inflammation-driven disease, (APC can present antigens to stimulate specific T cell receptors (TCRs), resulting in the activation and proliferation of T cells [53]. Often, T cells will release a series of inflammatory factors that further promote the inflammatory response under the presence of antigens. Common antigens include ox-LDL, as immunogens that activate the autoimmune system [54]. In addition to ox-LDL, heat shock proteins and peptides from some pathogens (HIV, HCV) can also act as antigens to induce atherosclerosis development via the APCs presentation activity [55, 56]. Interestingly, programmed cell death 1 ligand 1 (PDL1) on APCs interacts with PD1 and CD80 on T cells to inhibit T cell activation and reactivity to plaque-related antigens in the early stage of atherosclerosis [57]. Blocking this pathway will accelerate atherosclerotic progression [57-59]. In addition, the binding of CD137 ligand to CD137 promotes T-cell proliferation, leading to proatherogenic effects [60]. Moreover, OX40 ligands that interact with OX40 on T cells can promote T cell differentiation into TH1, TH2 and TH9 cells with proatherogenic effects [61]. All of the evidence indicates the involvement of T cells in atherosclerosis development. Hence, different APCs subtypes may also have opposing effects, given their capability to inhibit T cell activation and proliferation. For example, dendritic cells (DCs) in different subtypes play opposing roles in regulating the proliferation and differentiation of T cells [62, 63]. Under normal physiological conditions, CD11c^+^DCs are the dominant subendothelial DCs, which can induce the differentiation of Treg cells and exert anti-atherosclerosis effect [64]. When the Flt3 signaling pathway that regulates the maturation of CD11c^+^DCs was inhibited, a decrease in Treg cells concentration and atherosclerosis occurrence will be observed [64]. In contrast, CCL17 + DCs, another subtype of DCs, can prime antigen-specific T cell responses to increase the concentration of CD4 + T cells and then promote atherosclerosis by inhibiting Treg differentiation and promoting Treg apoptosis by regulating the Jak/Stat signaling pathway [65]. Often, differentiated, or undifferentiated T cells need the guidance of a range of chemokines in order to migrate to the lesion area which are demonstrated in Figure 4.

**Figure 5.**
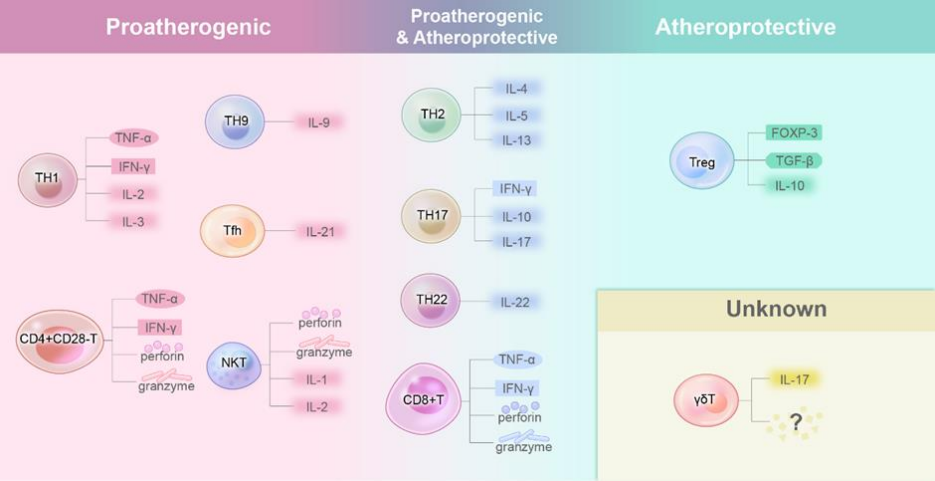
**Cytokine of T cell subsets and their effects on atherosclerosis**. T cells can elicit downstream responses in atherosclerosis by secreting several different cytokines, some of them are proatherogenic, such as TNF-α, IFN-γ, et al., while others are atheroprotective, such as IL-10. A class of T cells subtypes mainly secrete those above proaterogenic cytokines, so they have a proatherosclerotic effect. Treg are considered to have a protective effect on atherosclerosis, which may be related to its secretion of FOXP-3 and IL-10. Some other T cells subtypes may have both atheroprotective effect and proatherosclerotic effect at the same time.

Intriguingly, studies have found that the subtype of T cells determines their unique roles in the development of atherosclerosis [66]. Different T cell subsets express different genes and secrete different cytokines. It has been found that T helper 1(TH1) and T helper 2 (TH2) cells [67] are the primary T cell subsets deposited in atheromatous plaques. Differentiation of T cells to TH1 cells depends on the binding of APCs to the corresponding receptors (for example, CD40 [68], CD80 and CD86 [69]). In the studies using experimental animal models, blockade of these signals contributes to attenuation of atherosclerosis [70, 71]. The role of TH2 cells in atherosclerosis is controversial. Some clinical studies have found that individuals with high TH2 cell blood count levels are less likely to have early atherosclerosis [72, 73]. TH2 cells secrete various cytokines, among which IL-5, IL-4 and IL-13 are shown to be atheroprotective [74-77]. Interestingly, IL-4 and IL-13 are two cytokines having similar structure and function, but their atheroprotective effect is still under debate. Some studies indicate that IL-4 and IL-13 play an anti TH1 role in mice by promoting T cell polarization more toward TH2 cells [78-80], leading to an anti-atherogenic effect. In contrast, other studies demonstrate that high IL-4 and IL-13 production is related to hyperlipidemia [81], which may increase the risk of developing atherosclerosis. Interestingly, Kings suggests that IL-4 and IL-13 do not affect the development of hypercholesterolemia or angiotensin II-induced atherosclerotic lesions in Ldlr^-/-^ mice [82]. To this end, TH2 cells may have an enhanced atheroprotective effect as compared to their pro-atherogenic. However, the result remains inconclusive. Therefore, more thorough studies need to be conducted to explore the correlation between T cells and the development of atherosclerosis (Fig. 5).

##### 2.1.2.2 Natural Killer T (NKT) cells

NKT cells are a type of lymphocyte discovered in recent years, which are involved in the active and passive immune processes. It can express T cell receptors (TCRs) and receptor NK1.1. NKT cells can directly recognize antigens on the target cells' surface and can be activated by inflammatory factors such as IFN-γ. As inflammatory cells, NKT cells can activate downstream inflammatory responses by releasing inflammatory factors (TNF-α, IFN-γ, interleukins, et al.) after activation [83]. What’s more, NKT cells can also target CD1d on the cell surface and exert pro-apoptotic functions by secreting perforin and granzymes [83]. Based on the difference between TCRs expressed on the surface and whether CD1d is needed for maturity, NKT cells are categorized into NKT1, NKT2, and NKT3. Like T cells, NKT cells also have the selective expression of CD4 and CD8, which can be further divided into CD4NKT, CD4^-^CD8^-^NKT and CD8NKT. The development of CD4KNT and CD4^-^CD8^-^NKT is CD1d-dependent, and CD8NKT is CD1D-independent [84]. Li transplanted NKT cells lacking IFN-γ, IL-4, IL-21, perforin or granzyme B into NKT cell-deficient apoE ^-/-^ Jα18 ^-/-^ mice and found that only the mice lacking perforin and granzyme B showed a reduction in atherosclerosis, which demonstrates that CD4^+^NKT cells promote atherosclerosis mainly by expressing perforin and granzyme [85]. Since NKT cells participate in the inflammatory response mainly by secreting a variety of inflammatory factors mentioned earlier, Li et al. used DPPE-PEG350, a specific antagonist of CD1d, reduced MIP-1 and VCAM-1 expression and reduced plaque area [86]. At present, the specific role of NKT cells in the progression of atheromatous plaques remains elusive.

##### 2.1.2.3 B cells

According to the difference of CD5 expression on the surface of B cells, they can be mainly divided into two types: B1 and B2 cells. According to existing studies, they play opposite roles in the development of atherosclerosis.

B1 cells, on the one hand, exert anti-atherogenic effects by secreting IgM antibodies. After transplantation of B1a cells into splenectomized ApoE^-/-^ mice, a significant reduction in atheromatous plaques was observed [97]. Natural IgM antibodies secreted by B1 cells can specifically recognize oxidation-specific epitopes (OSE). Ox-LDL within OSE binding to IgM can block CD36 and SR-B1 receptor-dependent macrophages phagocytosis and reduce foam cell formation [98]. When IgM binds to phosphatidylcholine, it can activate complement C1q pathway-dependent endocytosis, promoting phagocytosis of macrophages to apoptotic cells and reducing local inflammation [99].

B2 cells, on the other hand, secrete a variety of Ig, of which IgE has been identified to have pro-atherogenic capacity. IgE secreted by B2 cells promotes the secretion of inflammatory factors by mast cells, enhances macrophages phagocyte ox-LDL, and accelerates the formation of foam cells [100]. At the same time, IgE could enhance cell membrane Na^+^/H^+^ exchange, resulting in the decrease of extracellular pH leading to cell apoptosis [101]. A study showed that using CD20 antibodies to knock out B2 cells from ApoE^-/-^ mice can reduce the atheromatous plaque area, demonstrating the pro-atherogenic ability of B2 cells [102].

**Figure 6.**
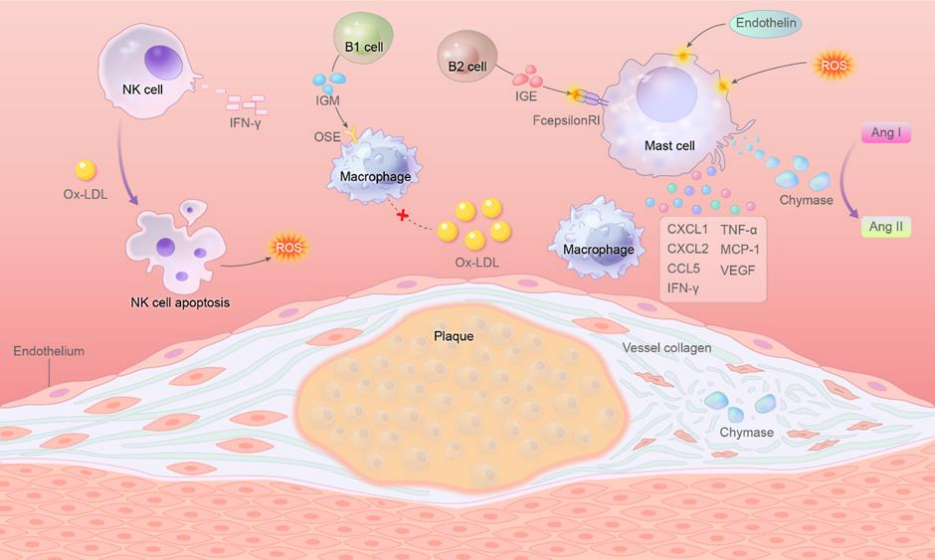
**NK, B and Mast cells can work synergistically in atherosclerosis development**. Inflammatory cells can be recruited to atherosclerosis sites by chemokines, and then activate and secrete several chemokines and inflammatory factors under the influence of complex molecular environment in the lesion site. These chemokines can further aggravate the infiltration of inflammatory cells. Inflammatory factors secreted by inflammatory cells can interact with each other to further promote the occurrence of inflammatory responses. For example, mast cells can secrete a series of chemokines to promote B cell recruitment, while B2 cell activation can secrete IgE to promote mast cell degranulation.

Thus, a series of enzymes, cytokines or other substances produced by NK, B and mast cells can allow them to recruit or activate each other, resulting in downstream inflammatory responses and tissue damage (Fig. 6).

### 2.2 Endothelial cells

Endothelium directly affects the selective penetration of substances in contact with blood contents at the vascular wall. In addition, endothelium plays an important role in the anti-coagulant and coagulation system. Physically, endothelium can separate platelets from extravascular collagen to avoid thrombosis formation at their contact site [127]. Endothelium regulates coagulation balance by secreting cytokines, including thrombomodulin (TM), tissue factor pathway inhibitor (TFPI), tissue-type plasminogen activator (t-PA) and heparan sulphate, to inhibit coagulation [128]. Meanwhile, endothelium also releases coagulant factors such as tissue factor (TF), thrombin receptors, von Willebrand factor (vWF) and plasminogen activator-inhibitor-1 (PAI-1) to achieve coagulation [129]. Activation of endothelial cells and changes in secreted cytokines dynamically regulate thrombus formation. Recent studies have demonstrated that endothelium can act as a sensor of shear stress and regulate the circulatory system's response [130]. Besides maintaining normal physiological functions, endothelium also contributes to disease development, such as local inflammation, by secreting a series of angiotensin, contractile and inflammatory factors. The anatomical localization of atherosclerosis, an inflammatory disease that occurs based on lipid accumulation, is located below the vascular endothelium. As a result, endothelium is the most direct site of involvement in the occurrence and progression of atherosclerosis.

Endothelial cells (ECs), a group of cell layers attached to the inner surface of the endothelium, have the function that regulates the communication between intra/extra-vascular substance and secretory capacity. Nitric oxide (NO) is one of the key secretions released by ECs. There are many subtypes of NOS in the human body. Among them, endothelial nitric oxide synthase (eNOS) and neuronal nitric oxide synthase (nNOS) have the anti-atherosclerosis effect, while inducible nitric oxide synthase (iNOS) can promote atherosclerosis [131]. ECs can release NO via eNOS [132] under stimulation (physical or chemical) or low shear stress. NO exerts physiological effects by activating the guanylate cyclase pathway(cGMP) on vascular smooth muscle or blood cells [133], such as inhibition of platelet aggregation [134], decreasing leukocyte adhesion [8] and smooth muscle relaxation. In addition, NO is also a reducing agent that can counteract the transition of LDL to ox-LDL and inhibit ROS generation [135]. The abnormal release of NO or the abnormal utilization of NO by the body may lead to the failure of related regulation, resulting in the development of atheromatous plaques [136]. On the contrary, iNOS is limited to blood vessels under normal physiological conditions. However, iNOS expression is upregulated in blood vessels when oxidative stress or inflammation occurs [137]. The irregularly over-expressed iNOS hijacked tetrahydrobiopterin (BH4) from eNOS, leading to a downregulation of the eNOS pathway [138]. eNOS is unable to transfer electrons from O_2_ to O_2 -_ due to the lack of BH4, which would also lead to oxidative stress in endothelial cells [139]. At the same time, during iNOS expression, the intermediate product can accelerate the oxidation of LDL [140]. NO can be produced long-term and in large amounts via the iNOS pathway, while excessive NO-generated peroxynitrite is also strongly oxidative [141]. When superoxide dismutase activity in the body is insufficient to process excessive peroxynitrite, those above processes become an important factor in the development of atherosclerosis.

The change of blood laminar flow status plays an important role in the occurrence of atherosclerosis. Shear stress refers to the longitudinal friction against the vessel intima during blood flow. It correlates with vessel thickness, blood flow velocity, and blood viscosity [142]. Shear stress under the physiological range (approximately 15 dyn/cm2), can promote the differentiation of endothelial progenitor cells into ECs, stimulate the antithrombotic function of ECs and inhibit the proliferation of ECs [143]. When shear stress is reduced, the apoptosis and pathological proliferation of ECs are enhanced, and a proinflammatory phenotype characterized by leukocyte adhesion is induced [143]. Previous studies suggest that shear stress can affect the development of atherosclerosis by affecting the alternative expression of microRNAs. For example, ECs under high shear stress can increase the expression of MiR-10a and play an anti-atherosclerotic role by inhibiting the IκB/NF-κB pathway [144]. Those miRs with atheroprotective effects include miR-10a, miR-19a, miR-23b, miR-101 and miR-143/145, which are all upregulated in high shear stress states. While in a low shear stress state, ECs promote atherosclerosis development by expressing miR-92a and miR-663 [143]. In addition, the types of shear stress also impact on atherosclerotic development differently. For example, after being treated with the unidirectional shear stress (USS), HUVEC cells can overexpress miR-21 to reduce ECs apoptosis and promote NO production via the eNOS pathway [145]. However, HUVEC cells increased VCAM-1 and MCP-1 expression by elevating miR-21 expression when exposed to oscillating shear stress (OSS) [146]. Upregulated expressed miR-21 also downregulates the expression of peroxisome proliferator-activated receptor-α (PPAR-α), which enhances the inflammatory response [147]. To sum up, abnormal shear stress can lead to abnormal ECs function and trigger atherosclerosis.

**Figure 7.**
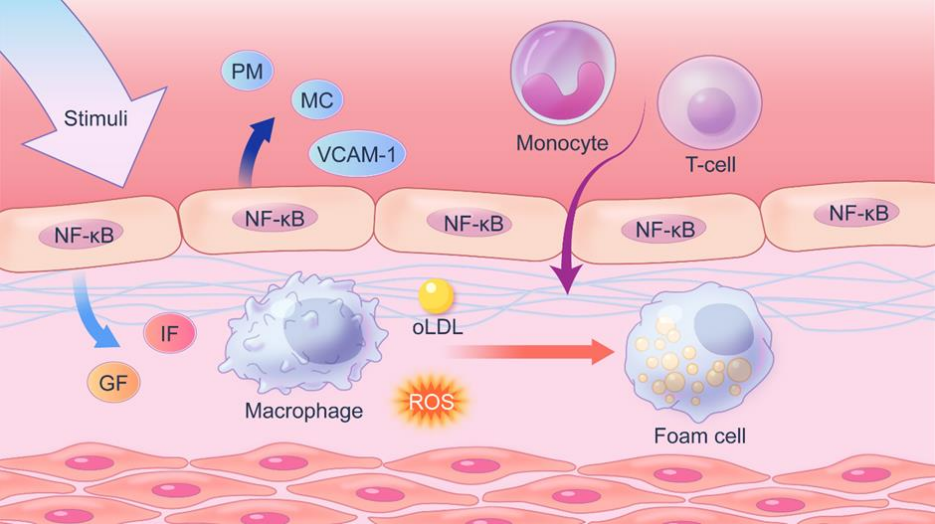
**Endothelium secrete cytokine promotes monocyte adhesion and T cell homing**. Response-to-Injury Hypothesis suggests that nuclear factor NF-κB of ECs will activate and promote the transcription and secretion of several chemokines and cytokines as a result of suffering from stimulation, which is the most important factor in atherosclerosis development. Chemokines can promote the recruitment of inflammatory cells and induce monocytes to differentiate into macrophages to eventually form foam cells. The lipid core based on foam cell constitution is the most important structural basis of atherosclerosis lesion.GF=Growth Factor; IF=Inflammation Factor; PM= Prothrombotic Mediator; MC=Membrane chemokines; ROS= Reactive Oxygen Species.

Regarding the pathological development of atherosclerotic plaque, the response-to-Injury Hypothesis, introduced by Ross, suggested that the loss of integrity in the endothelium is a typically early characteristic of atherosclerosis [148]. The hypothesis suggests that mechanical (chronic hemodynamic abnormal) or chemical stimulation (harmful components in cigarettes, hyperglycemia, hyperhomocysteinemia [149], et al.) can lead to the intimal injury, resulting in the apoptosis and injury of ECs. Apoptotic ECs further release inflammatory factors, allowing circulating monocytes to selectively migrate to the sub endothelium and differentiate into macrophages at the lesion region, resulting in the formation of macrophage foam cells. For instance, chemical stimuli such as oxidized lipoproteins, inflammatory substances (IL-1, endotoxin, TNF, et al.), or advanced glycation end products (AGEs), can activate the NF-κB pathway and allow endothelial cells to release a number of downstream factors, including prothrombotic mediators (TF, vWF, PAI1), membrane chemokines and adhesion molecules (VCAM-1) [150], promoting the recruitment of monocytes and homing of T-lymphocytes to the damaged site. The recruited immune cells further enhance the persistence of chronic inflammation and aggravate the disease progression (Fig. 7). Meanwhile, after the endothelial injury and abnormal NO synthesis, platelets adhere to the damaged site and release platelet-derived growth factors, resulting in vascular smooth muscle proliferation, also known as fibromuscular plaque production [151]. After binding to guanylate cyclase (sGC) on cell membrane, endogenous NO can reduce intracellular Ca2+ concentration through cGMP pathway, followed by an inhibition on platelet aggregation. On the contrary, platelet adhesion increased when abnormal NO production occurs [152]. Growth factors (PDGF-b [153]) secreted by macrophages and ECs can also enhance vascular smooth muscle proliferation and produce fibromuscular lesions [154]. Together, those simultaneous responses after the endothelial injury contribute to the lipid stripe creation and plaque expansion. It is worth mentioning that endothelial injury in animal models, including balloon dilatation artery to produce vascular endothelium mechanical damage and high lipid diet to induce local atherosclerosis, are under the guidance of this hypothesis.

### 2.3 SMCs

**Figure 8.**
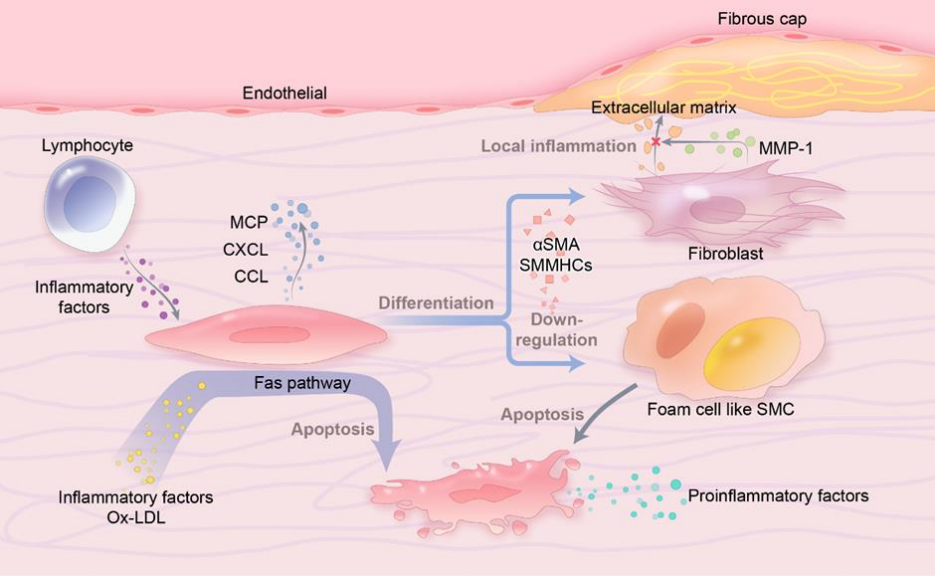
**Phenotypic changes of smooth muscle cells in atherosclerotic plaques**. In atherosclerotic plaques, contractile smooth muscle cells differentiate into secretory smooth muscle cells, which results in accelerated plaque formation and reduced plate stability.

The stability of atheromatous plaques is closely correlated with the content of SMCs within the lesion [155, 156]. It is believed that SMCs can provide stable fibrous caps and secrete extracellular matrix [157] in matured atheromatous plaques to improve plaque stability. Interestingly, studies have suggested that SMCs may play an opposite role at the early stage of atherosclerosis. During the initial phase of plaque development, the extracellular matrix produced by SMCs can bind with apolipoproteins due to the ionic interaction between basic amino acids in apoB100 and negatively charged sulphate groups on the proteoglycans [158], which aggravates the accumulation of lipids at sub-intima, as a result provides the structural support for atherosclerotic plaque. In addition, stimulated by inflammatory factors secreted by lymphocytes or other neighboring cells, SMCs secrete chemokines (MCP-1, CCL5, CXCL1) that allow monocyte recruiting to the sub-endothelium [159-162]. With the growth of lesion size, differentiation of SMCs into several subtypes may accelerate this progress. For instance, SMCs differentiate into fibroblasts, contributing to the progression of atherosclerosis, where transcription factor 21 (TCF21) plays an important role in this process [163]. Studies have found that TCF21 is highly expressed in fibroblasts differentiated from SMCs. There are two aspects of those fibroblasts that affect atherosclerosis. On the one hand, the extracellular matrix secreted by fibroblasts enlarges the fibrous cap, increasing plaque volume. On the other hand, by secreting MMP-1, fibroblasts dissolve the extracellular matrix and elicit downstream inflammatory responses [164]. In addition, the interaction between different inflammatory factors secreted by different phenotype SMCs also impacts the morphology of atheromatous plaques. For example, foam cell-like SMCs form a necrotic core after apoptosis, and the leaking proinflammatory substances can promote a healing response to generate a fibrous cap [165]. Overall, the transition of contractile SMCs to secretory SMCs and their migration to the vessel intima is an important driver for the development of atherosclerosis. Because secreted SMCs have active proliferation and enhance proteoglycan synthesis ability compared to contracted SMCs. Among the transition of SMCs phenotypes, decreased expression of some typical proteins, such as α-smooth muscle actin (α-SMA) and smooth muscle myosin heavy chains (SMMHCs), leads to the changes in SMCs function [166]. Recent studies have found that integrins are important factors in inducing the dedifferentiation of SMCs, and down-regulated expression of integrin α1β3, α7β1 and α8β1, resulting in the conversion of contractile SMCs into secretory SMCs [167]. Taken together, different phenotypes of SMCs play different roles in the development of atherosclerosis (Fig. 8).

Apart from the contribution of their ECMs to the structural support for the atherosclerotic lesion, apoptosis of SMCs is also involved in the development of atherosclerosis. Some common stimulators in the development of atherosclerosis, such as inflammatory factors (e.g., IFN-γ, TNF-α, et al.), ox-LDL, and high levels of NO, can induce apoptosis of SMCs through the Fas (CD95) signaling pathway [168]. IL-1β secreted by apoptotic SMCs can elicit an inflammatory response in normal SMCs nearby. It has been found that apoptotic SMCs can be phagocytosed and removed by normal SMCs; however, the phagocytic ability is reduced under hyperlipidemia [169]. The ineffective clearance of apoptotic SMCs and the spread of the inflammatory response further contribute to further progression of atherosclerosis.

However, the current knowledge in SMC is still limited. Hence, further studies on related inflammatory or non-inflammatory mechanisms for SMCs can help researchers better understand atherosclerotic progression, providing a new therapeutic strategy for the treatment of atherosclerosis.

### 2.4 Platelets

Unlike other inflammatory cells, the key function of platelets is to help the formation of a thrombus. It is believed that platelets participate in thrombosis after atherosclerosis plaque rupture and lead to subsequent hypoxia events. However, recent studies have confirmed that platelets also contribute to the progression of atherosclerosis.

Changes in blood flow shear forces trigger activation of platelet surface receptors, allowing platelets to bind to the local extracellular matrix [170]. When vascular endothelial damage occurs locally, platelets quickly activate and adhere to the damage region, following by the expression of downstream signals to promote other inflammatory cells response. During this process, glycoproteins on platelet membranes, such as GPIb, bind to the A1 domain of von Willebrand Factor (VWF) from the extracellular matrix. Then calcium mobilization leads to the reorganization of the intracellular cytoskeleton of platelets, altering the shape of platelets into a spherical shape with pseudopods protruding, which further enhances adhesion [171]. In addition, toll-like receptors (TLRs), which are expressed on platelets to recognize pathogens and induce platelets exert immune resistant properties, have been suggested to be involved in the development of atherosclerosis. For instance, LDLR^-/-^ mice lacking TLR2 are found to have reduced macrophage recruitment and reduced atherosclerotic lesions [172]. However, the specific involvement of TLRs in the disease process remains unclear and needs further investigation.

Furthermore, when platelets adhere to the endothelial or extracellular matrix, p-selectin is expressed and binds to p-selectin Glycoprotein Ligand 1 (PSGL-1) on leukocytes, resulting in platelet-leukocyte aggregation [173]. Aggregation and activation of leukocytes to the injured area result in downstream inflammatory factors secretion [173].

Activated platelets also contribute directly to local inflammation by secreting cytokines and chemokines. Il-1 β and CD40L are two of the most important cytokines, which induce platelets-bound endothelial cells to secrete chemokines (CCLs) and adhesion factors (ICAM-1, VCAM-1), promoting the recruitment of immune cells consequently [174]. Activated platelets can also directly secrete chemokines, such as CXCL4 and CCL5, and lead to recruitment of immune cells [175]. In addition to the above-mentioned pathways, platelets have recently been found to regulate suppressor of cytokine signaling 3 (SOCS3), thereby promoting the differentiation of macrophages into inflammatory phenotypes and promoting the progression of atherosclerosis [176].

A review of existing studies suggests that antiplatelet agents have beneficial effects on the progression of atherosclerotic plaque itself, in addition to reducing the risk of thrombosis. However, most of the current studies on the relationship between platelet and atherosclerosis focus on the formation of thrombosis. The mechanism of platelet in atherosclerotic development requires more investigation.

## 3. Advances in molecular and cellular-based therapy

Given cellular response after the endothelial injury initiating the development of atherosclerosis, hypothetically, interference within cellular responses such as inhibiting EC apoptosis, restricting immune cells recruitment, downregulating SMC migration, and preventing foam cells formation, can potentially slow and prevent the progression of atherosclerosis. Hence, recent studies have been exploring the possibility of using molecular and cellular-based technologies such as gene editing or nanotechnologies, for atherosclerosis treatment.

### 3.1 Gene editing

Gene-editing technology is an important means of modern scientific research. To date, there are generally three generations of gene editing technologies, namely ZFNs, TALENs and CRISPR/Cas9.

Zinc-finger protein (ZFP) was discovered by Miller in the Xenopus transcript TFIIIA [177]. ZFNs technology is to fuse ZFP and FokI endonucleases. ZFP recognizes specific DNA sequences and FokI cleave identified DNA chains, resulting in the break of double-strand. Ultimately, knockout or knock-in of the target gene is achieved by the natural repair mechanism within the cells. Once proposed, ZFNs have attracted the attention of researchers. In the field of cardiovascular diseases, the construction of animal models, such as pig models with PPARγ gene knockout [178], has helped the research in the related disease studies. However, ZFNs are complex in design and prone to off-target, resulting in limited downstream applications.

Transcription activator-like effectors (TALEs), originally isolated from Xanthomonas [179], can bind to the promoters of host cell genes and activate gene expression. This technique combines TALEs and FokI and has been used for target gene editing (also known as TALENs). Through the modification of the 12^th^ and 13^th^ amino acids in the proteins, TALEs can recognize special base pairs on DNA. Then knockout or knock-in of genes could be achieved after cleavage of DNSs via FokI. Compared to ZFNs, TALENS has reduced costs, improved targeting efficiency and higher efficiency. However, when TALENs that recognize more base pairs are designed, extended long peptide chains may lead to immunogenicity. What’s more, TALENs are not suitable for knock-in gene fragments.

In 2012, Doudna and Charpentier proposed a method of gene editing using CRISPR-Cas9 [180]. Since then, attempts have been made using CRISPER-Cas9 to treat many diseases. CRISPR-Cas9 can engineer pathogen genomes for therapeutic purposes and induce therapeutic mutations in host tissues [181]. The basic principle of CRISPR-Cas9 is derived from the adaptive immune system of microorganisms, which can specifically cut off the gene of the virus, resulting in inactivation. The purpose of knock-in genes can be achieved by introducing target DNA fragments. Seeger found that CRISPR-CAS9 technology can be used to remove hepatitis B covalently closed circular DNAs (cccDNAs) with excellent therapeutic potential [182]. Wu used CRISPR-CAS9 technology to introduce target correction genes into spermatogonial stem cells and cured disease-causing Crygc mutation in mice by combining eggs to produce offspring with corrected phenotypes [183]. Liu used CRISPR-Cas9 technology to exercise the mouse ACE2 gene by targeting the third exon near the transcriptional start codon and the 18^th^ exon downstream of the mice ACE2 gene [184]. This mice model showed a predisposition to vascular lesions due to reduced ACE2 expression. Compared with traditional gene-editing technology, CRISPR-Cas9 is simple, efficient, and low-cost [185]. Since CRISPR-Cas9 originates from the immune system of bacteria, it has inherent advantages in targeting diseases caused by viruses. For example, CRISPR-Cas9 treatment can inhibit cell proliferation and reduces viral load in EBV-infected Burkitt's lymphoma cell lines [186].

Given that some risk factors for atherosclerosis are associated with genes, such as hypercholesterolemia, diabetes and hypertension, abnormal physiological functions that occur due to mutations in related regulatory genes, CRISPR-Cas9 can be potentially applied to eliminate the risk factors, resulting in the prevention of atherosclerosis. For example, Zhao successfully generated Ldlr-E208X mutant mice mimicking familial hyperlipidemia (FH) using CRISPR-CAS9 [187]. They used adeno-associated virus (AAV) as a vector, loaded with the E208X mutation (GAG > TAG) in the Ldlr gene, and knocked it into mice zygotes. The target gene (liver-specific thyroxin-binding globulin promoter) was further introduced into neonatal Ldlr-E208X mutant mice by AAV to treat hyperlipidemia in mice [187]. In another example, Ding used AAV carry NGG sequence, resulting in a frameshift mutation and knockout of PCSK9 gene [188]. The reduction in plasma PCSK9 levels and cholesterol levels can decrease the risk of developing atherosclerosis. Similarly, Guo used CRISPR-CAS9 technology to excise 37 bases in the hamster Apoc3 gene and inactivate it [189]. The decrease in ApoC3 expression increases lipoprotein lipase activity, which in turn plays a role in lowering blood lipids and preventing atherosclerosis.

In addition to using viruses as vectors, researchers have successively discovered that it is possible to use vectors such as liposomes or polymer materials for CRISPR-Cas9 in recent years. Cho used lecithin nano-liposome particle as a CRISPR/Cas9 complex delivery system for treating type 2 diabetes by downregulating the expression of enzyme dipeptidyl peptidase-4 (DPP-4) gene, which can block the degradation of glucagon-like peptide-1 (GLP-1) by DDT-4 [190]. Their result showed improved stability GLP-, leading to enhanced insulin secretion. It eventually led to a decrease in blood glucose in mice. Chin used a scaffold made of polyDOPA-melanin (pDOPA) to adsorb the liposome-encapsulated CRISPR-Cas9 system, overcoming the low cellular targeting ability of traditional CRISPR-Cas9 therapy [191]. Chen prepared thin glutathione (GSH)-cleavable covalently crosslinked polymer coating as an alternative AAV shell in the CRISPR-Cas9 system, which has the advantages of high safety and small particle size [192].

Although CRISPR-Cas9 technology has great potential in the treatment of atherosclerosis, the long-term impact of the CRISPR-Cas9 is still under investigation. Also, CRISPR-Cas9 technology is dominantly used in preclinical animal models, due to the potential risk of an unpredictable consequence of the gene mutation and species barriers. More studies need to be conducted before the clinical practice of CRISPR-Cas9 technology.

### 3.2 Nanocarrier drug delivery

Nanocarrier technology is another popular approach for atherosclerosis treatment. Due to their nano size (normally under 200nm), they can bypass lysosomes, reducing the loss of bioactive components within the nanocarriers caused by hydrolytic enzyme and lysosomal degradation. The therapeutic function of nanocarriers depends on the bioactive components encapsulated within nanocarriers. Drugs, miRNA, among other pharmaceutical factors, are the common bioactive ingredients that have been loaded with nanocarriers for atherosclerosis treatment.

Various drugs, including statins, aspirin, fenofibrate and evolocumab, have been used clinically to treat atherosclerosis. In addition to these traditional drugs, some drugs originally used to treat other diseases have also been found to have treatment efficacy on atherosclerosis. For example, rapamycin can inhibit macrophage proliferation and neovascularization, but it has side effects leading to hyperlipidemia and interstitial lung disease [193]. Fumagillin and its analogue TNP-470 are effective drugs that inhibit angiogenesis; however, the neurotoxicity limits their usage in atherosclerosis treatment [194, 195]. Celastrol can play an anti-atherosclerotic role by inhibiting LOX-1, but it has not been put into anti-atherosclerosis clinical application due to the cytotoxicity and low bioavailability [196, 197]. Thus, researchers have focused on developing nanocarrier delivery systems to improve the therapeutic effect and reduce side effects of drugs.

Moreover, with the progression of molecular biology research, the correlation between RNAs and atherosclerosis development has been identified. Therefore, in addition to pharmaceutical drugs, non-traditional drugs such as nucleic acids (siRNAs, shRNAs, miRNAs, et al.) have also been encapsulated into advanced nanocarrier systems for atherosclerosis treatment.

**Figure 9.**
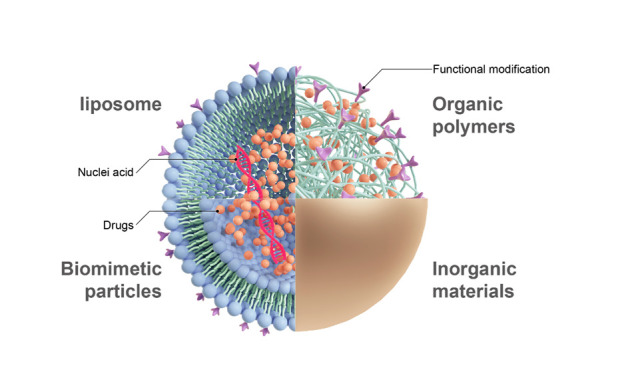
**Four types of nanocarriers are commonly used**. There are four types of nanocarriers in common use. They are liposomes, Organic Polymers, Biomimetic Particles and Inorganic Materials.

Often, the materials used in nanocarriers to encapsulate drugs are fat-soluble materials, which has improved solubility within cell membranes and the potential for modification with targeting molecules. After being modified by some special molecules, such as mannose or P-selectin, nanocarriers acquired higher targeting ability to cells expressing the corresponding receptor. To date, the most popular wrapping materials applied to nanocarriers include liposomes, cell membrane components, organic polymer materials and inorganic materials (Fig. 9).

#### 3.2.1 Liposomes

When molecules with both hydrophilicity and hydrophobicity ends are dispersed in water, the hydrophobic ends between those molecules tend to aggregate with each other and exposing the hydrophilic head to water, the closed vesicles with bilayer molecular structures formed are called liposomes. As one of the most popular nanocarriers, liposomes have been widely applied in laboratory research as well as clinical applications. Dozens of liposome drugs have been successfully marketed or in different clinical research stages, such as Caelyx, amphotec, et al. Liposomes are presumably divided into three parts, a hydrophilic head, a hydrophobic tail, and an intervening connecting part. To meet specific functional requirements, PEG or antibodies have been used to modify liposomes with enhanced permeability, improved retention effect, EPR effect, or specific targeting ability. The properties of liposomes can be altered via the head, tail, and connection site, with great versatility. For example, compared with only one single quaternion-modified liposome head, use of arginine-modified heads can significantly increase the transfection effect of liposomes and lysosomal escape [198]. Koynova found that the transfection efficacy of liposomes increased correspondingly as the tail length and unsaturation increased [199]. Kawakami synthesized galactosylated cholesterol derivatives and found lower cytotoxicity and higher transfection efficiency than cholesterol tails [200].

The use of liposomes in atherosclerosis treatment is currently in the laboratory stage. Based on cellular involvement in atherogenesis, high specificity liposome drugs have been developed. Liposomes modified by H18/7, a specific antibody to E-selectin, can specifically bind E-selectin on the ECs at the inflammation location and reduce the inflammatory response [201]. Using anti-VCAM-1 antibody-modified liposomes, equipped with cyclopentenone prostaglandins (CP-PGs), can target plaque areas and reduce the inflammatory response [202]. Lectin-like LOX-1 is demonstrated to be upregulated in plaque areas and can play a pro-atherogenic role after binding to ox-LDL [203]. Injection of anti-LOX-1 antibody-modified liposomes blocked downstream processes in ECs and macrophages, demonstrating an anti-atherogenic effect [204]. In addition, directed knockdown of VCAM-1 using ECs targeted liposome delivery of siRNA inhibited downstream inflammatory responses and inhibited plaque formation [205]. Mocanu used P-selectin-targeted liposomes containing RAGE-shRNA on ApoE^-/-^ mice, and a reduction in the atheromatous plaque size has been observed due to the downregulation of RAGE protein expression [206]. In another study, Lu used anti-miR155 loaded liposomes target macrophages, which led to a downregulation in the ROS production and ox-LDL uptake [207]. The above examples suggest that liposomes can deliver traditional chemical drugs and novel therapeutic factors such as proteins and nucleic acids for atherosclerosis management.

The greatest advantage of liposomes is their high safety because of the low immunogenicity and the property of being easily metabolized. However, because the maintenance of liposomes morphology depends on hydrophobicity, liposomes are less stable and prone to fragmentation. What’s more, liposomes are also unable to carry large molecules, which limits their clinical applications.

#### 3.2.2 Biomimetic particles

Compared with other nanocarriers, the biomimetic particle has many advantages such as improved biocompatibility, high specificity, low immunogenicity, and easy preparation. The process of preparing biomimetic nanoparticles usually involves three steps. First, cells are lysed using hypotonic fluids, and cell membranes are further extracted by centrifugation separation. Most of the studies use circulating blood cells, such as erythrocyte, leukocytes, and platelets, as the cell source. Cell membranes derived from stem cells, tumor cells and bacteria also have been used. After the separation of cell membranes, the drug-laden nanoparticle particles are prepared. Finally, drug-loaded nanoparticles are encapsulated by the cell membrane. Encapsulation normally utilizes the electrical attraction between the nanoparticles and membranes due to the charge difference [208] or ultrasound stimulation [209]. Electroporation [210] is another approach to allow nanoparticles to enter microvesicles through holes in the cell membrane. Physical extrusion can also wrap the cell membrane around the microparticles. Different from electroporation, it may destroy the membrane structure [211]. In addition to encapsulating drugs within cell membrane vesicles, the method of combining nanoparticles with ligands outside cell membrane vesicles to transport drugs has also been developed with proven feasibility [212].

Because some highly expressed cytokines during atheromatous plaque formation have a recruiting effect on circulating cells, nanovesicles prepared using these circulating cell membranes have the advantage of high selectivity. Platelet membrane-coated nanoparticles (PNPs) are a classical option based on platelets' properties to adhere to atheromatous plaque areas. When atherosclerosis occurs, local inflammation activates platelets adhesion to plaque [213]. Based on this principle, Song used platelet membranes to encapsulate the PLGA core load with rapamycin and applied it to ApoE ^-/-^ mice [214]. They found a reduction of atheromatous plaque area and reduced systemic side effects caused by the application of rapamycin. Ma designed a Platelet Mimicking nanoconstruct (PM-PAAO-UCNPs) containing UCNPs and the Ce6 photosensitizer for accurate localization of plaques and noninvasive PDT of atherosclerosis [213]. Cell membrane-derived envelopes in this study increased the affinity of nanoparticles to macrophages. It can produce ROS after irradiation with near-infrared light, which induces apoptosis of foam cells and reduces atheromatous plaques' area.

Different from platelet membranes, erythrocyte-derived cell membranes have an immune escape effect due to the presence of CD47 on their surface [215]. Wang assembled RBC-NPs containing PLGA core loaded with rapamycin and erythrocyte cell membranes [216]. They found that the area of atheromatous plaque was reduced after the injection treatment to ApoE ^-/-^ mice. In this study, RBC-NPs were accumulated at the plaque area due to the local abnormal blood flow, then rapamycin from the particles promoted anti-inflammation, anti-migration, anti-proliferation and activated autophagy effects by inhibiting the mTOR pathway [216]. Compared with platelet-derived cell membranes, erythrocyte-derived cell membranes are highly biocompatible. However, the plaque targeting ability is insufficient.

In addition to the advantages of the above two cell membranes, immune cell-derived cell membranes may have additional functions according to their sources. Gao created ROS-responsive nanoparticles using nanoparticles centred on oxidation-sensitive chitosan oligosaccharide (Oxi-COS) loaded with atorvastatin and encapsulated with macrophage membranes [217]. These NPs cleave and release atorvastatin after encountering ROS. In addition to releasing drugs, receptors on the encapsulated macrophage membrane can also neutralize inflammatory factors, inhibit foam cell formation and block downstream inflammatory responses.

The greatest advantage of cell membrane-derived nanocarrier systems is the high biocompatibility and targeting ability because of their natural origin. Although more research efforts have been devoted to cancer treatment, it is undeniable that their potential is significant for cardiovascular diseases.

#### 3.2.3 Organic polymers

Organic polymeric material combined with drugs or proteins for therapeutic applications is also another popular approach to fabricating nanocarriers. It can bring targeting ability to drugs and improve their bioavailability or endow them with sustained-release capacity. Beyond that, encapsulation of polymer materials can also protect drugs from premature hydrolysis or rapid metabolism, resulting in a prolonged therapeutic effect [218]. At present, some polymers have been investigated, including poly (ethylene glycol), poly (vinylpyrrolidone), poly (l-lysine), poly (l-glutamic acid), poly (malic acid), poly (aspartamides) and dextran (α-1,6-polyglucoses) [219]. In addition, by altering the shape of the polymer, the physicochemical properties of the polymer can be manipulated and applied to different scenarios. For example, Winter used fumagillin loaded αvβ3-integrin targeted paramagnetic perfluorocarbon nanoparticles to inhibit angiogenesis from treating atherosclerosis in rabbits [220]. Yu used mannose to modify pH-responsive polymeric micelles and target macrophage mannose receptor CD206 [221]. Cargos (siRNA) released from the micelles inhibited cyclophilin-B gene expression and exerted an anti-atheromatous plaque effect. In another study, Dou used β-cyclodextrin load with rapamycin and found that it could target and inhibit mTORC1, resulting in slow progression of atherosclerosis [222]. Meanwhile, Chnari reported nanoscale anionic macromolecules based on amphiphilic scorpion-like macromolecules with a lauryl chloride-mucic acid hydrophobic backbone and poly (ethylene glycol) shell specifically adsorbed LDL and low-grade ox-LDL [223]. This polymer material can reduce blood lipid levels and exert antiathero-sclerosis function without additional drugs. Allen used PEG-b-PPS load with hydrophobic molecule celastrol, a NF-κB inhibitor, to treat with ldl^-/--^mice. Reduction in the number of inflammatory cells and atheromatous plaque area were observed [224].

Before being put into use, the safety, biocompatibility, and degradability of organic polymeric nanoparticles still require long-term systematic evaluation. Long-term in vivo application of organic polymer materials, such as PEGs, may lead to antibody production which result in reduced therapeutic effect and allergic reactions [225]. In addition, the large-scale production of organic polymeric nanoparticles is costly due to their structural complexity. Therefore, before a mature organic polymeric nanoparticles drug carrier system is approved for clinical application, it faces more challenges than traditional drugs.

#### 3.2.4 Inorganic materials

The construction of nanocarriers using inorganic materials is also an important research direction. Among them, very small superparamagnetic iron oxide particles (VSOP) have been proven to be useful for atherosclerosis MRI diagnosis in human trials. Using citric acid to encapsulate VSOP, Wagner discovered the system targeted macrophages and calcified microvesicles in the plaque, which can be used as a contrast agent for detecting atherosclerosis via MRI [226]. Apart from VSOP, gold nanoparticles are another popular material due to their excellent biocompatibility. Rizwan explored the anti-atherosclerosis therapeutic potential of gold particles and found that AuNPS can inhibit NF-κB activation within macrophages exert anti-atherosclerotic efficacy by inhibiting foam cell formation [227]. Liu used fluorescent magnetic nanoparticles to detect cytokines in atherosclerosis, which has high application potential [228]. Metal materials NPs often have unique advantages in stable properties, small and uniform particle size. However, unlike the research progress in the field of oncology, there are relatively few studies of metal materials in atherosclerosis, and most of them are limited to imaging diagnostic applications only. Chandramouli proposed that VSOP can be guided to plaque area by magnetic fields, which could be utilized to eliminate plaques using high temperature produced under rapidly switching magnetic fields [229], but further research is still lacking. The use of metal materials in oncology therapy is enlightening, but how to utilize the physical and chemical properties of metal materials before putting them into atherosclerosis treatment study is currently the first issue that researchers need to figure out.

Although nanotechnologies possess significant potential in atherosclerosis treatment, certain aspects need to be considered. Firstly, the bioactive ingredients encapsulated within nanocarriers should be effective with limited side effects. In addition, the materials used as nanocarriers need to be biocompatible and biodegradable. More importantly, biomaterials should have the capability to be modified with specific molecules to achieve targeted and precision delivery. Also, in order to reduce the immune responses, the nanocarrier should be able to incorporate certain immune recognition proteins, to bypass the immune cell detection.

## 4. Future perspective and conclusion

Atherosclerosis and its associated pathogenesis have been extensively studied. Chronic inflammation induced lipid accumulation is one of the natural characteristics of atherosclerosis. With the progressing understanding of atherosclerosis pathogenesis, the key roles of various immune cells have been identified. However, due to the diversity of inflammatory cells and their secreted inflammatory factors, many questions remain elusive. For example, the physiological function of some T cell subset in atherosclerosis. In addition, multiple cytokines secreted by several cells are involved in the development of atherosclerosis. The effect of these cytokines on atherosclerosis progression may be paradoxical and limit their application as a target for the treatment of atherosclerosis. More importantly, due to the complexity of atherosclerosis progression, the same physiological reaction may have opposite effects at different stages. For example, the fibrous cap is conducive to plaque stability in the late stage of the disease, but increases plaque volume in the early atherosclerotic stage. Currently, limited options are presented for researchers to fully understand the complex pathological development of atherosclerosis. The intensive use of animal models or human trials raises ethical concerns. Also, the in vitro model is inadequate to mimic the pathological progression. Therefore, the recent development of arterial organoid models would be an advanced tool to help us understand the mechanisms of atherosclerosis at the organ level. Organoid models are constructed by embedding specific stem or progenitor cells into matrigel or other appropriate extracellular matrixs with additional specific growth factors that allow cells to differentiate and proliferate into organ-like structures. Organoids present certain functions and physiological similarities to organs, widely used in the tissue engineering field to study the developmental and pathological progression of organs and diseases. Although organoid models are currently widely used in the field of oncology research, Wimmer pioneered the construction of arterial organoid models to study diabetic vascular lesions, which provided a breakthrough for the study of atherosclerotic diseases [230].

In recent years, the status of miRNAs in the progression of atherosclerosis has been gradually recognized. miRNAs can be released by autocrine or paracrine methods, participating in the advancement of atherosclerosis by regulating the expression levels of genes. Similar to the various cytokines mentioned above, miRNAs are also divided into pro-atherosclerotic and anti-atherosclerotic types. For example, miR-10a and miR-155 inhibit the inflammatory response and the expression of adhesion factors. In contrast, miR-21 can promote atherosclerosis by promoting immune cell adhesion [146] or increasing peroxide production [147]. Exosomes are microvesicles composed of homologous lipid bilayers of cell membranes and with a diameter under 150 μm. Exosomes can carry and release miRNAs, and relevant research has become a hot topic in the atherosclerosis research field in recent years. The goal of exosome research is to promote beneficial exosome secretion or reduce the production of unwanted exosomes. In vitro studies have confirmed that the specific drugs can be used to change the secretion of exosomes, resulting in an anti-atherosclerotic effect. At present, the commonly used anti-atherosclerotic drugs in clinical practice, in addition to directly changing the protein expression of cells, also regulate the transcription of downstream cells through the exosome pathway. For instance, simvastatin inhibited monocyte secretion of exosomes containing miR-150, leading to elevated c-myb in endothelial cells and inhibited endothelial migration, which restrained the development of atherosclerosis [231]. Furthermore, Shi found that paeonol could promote miR-233 secretion to reduce endothelial NLRP3 pathway-related inflammation [232]. In addition to the natural exosomes, engineered exosomes using exosomes as a carrier to deliver molecular drugs are another research direction in the field. Banizs loaded endothelium-derived exosomes with siRNA using electroporation and observed an anti-inflammation effect on endothelial cells [233]. Despite the advantages of high histocompatibility of exosome drug-loading systems, their disadvantages, such as low drug-loading efficiency and easy clearance from the liver, limit their downstream clinical translation.

In conclusion, the understanding of atherosclerosis has far exceeded the most superficial cause of lipid accumulation that has been known in the past. Studies have extended our understanding of atherosclerosis in response to deeper systemic causes research, such as systemic metabolic disorders, familial blood pressure abnormalities, or increased susceptibility to diseases caused by intestinal bacteria change. At the same time, they also provide some new perspectives on the prevention and treatment of atherosclerosis. These new perspectives, combined with new therapy technic, will provide ceaseless new ideas in atherosclerosis research.
